# Strategies to Improve Inpatients' Quality of Bowel Preparation for Colonoscopy: A Systematic Review and Meta-Analysis

**DOI:** 10.1155/2019/5147208

**Published:** 2019-05-02

**Authors:** Paraskevas Gkolfakis, Georgios Tziatzios, Ioannis S. Papanikolaou, Konstantinos Triantafyllou

**Affiliations:** Hepatogastroenterology Unit, Second Department of Internal Medicine-Propaedeutic, Research Institute and Diabetes Center, Medical School, National and Kapodistrian University of Athens, “Attikon” University General Hospital, Athens, Greece

## Abstract

**Background and Aims:**

Inpatients' bowel preparation before colonoscopy is frequently inadequate, and various interventions have been investigated to improve it, so far. We aimed to evaluate the efficacy of various interventions to improve inpatients' colon preparation quality.

**Methods:**

We systematically reviewed the literature for publications on interventions aiming to improve the quality of inpatients' colon preparation until June, 2018. Significant heterogeneity—measured with *I* ^2^—was detected at the level of *P* < 0.1. Adequacy rates were measured using inverse variance, and the size effect of different interventions was calculated using random effects model and expressed as odds ratio (OR).

**Results:**

Seventeen studies enrolling 2733 inpatients were included. Overall, 67% (60-75%) of the participants achieved adequate colon cleansing (*I* ^2^ = 97%; *P* < 0.001). In six studies assessing the impact of educational interventions to patient/physician/nurse vs. no intervention, adequate bowel preparation was achieved in 77% (62-91%) vs. 50% (32-68%) of the patients (OR (95%CI) = 3.49 (1.67-7.28), *P* = 0.0009; *I* ^2^ = 74%; *P* = 0.002). Ten studies examined variations (qualitative and/or quantitative) in bowel preparation regimens with adequate preparation detected in 71% (60-81%) of the participants, and a single study examined the administration of preparation through an esophagogastroduodenoscope, resulting in adequate prep in 71% of the patients.

**Conclusions:**

Despite several interventions, only two-thirds of inpatients achieve adequate colon preparation before colonoscopy. Educational interventions significantly improve inpatients' bowel preparation quality.

## 1. Introduction

Hospitalization compared to the ambulatory setting is associated to an almost twofold higher risk of failed bowel preparation before colonoscopy, while the rate of inpatients with adequately prepared colon does not exceed 50%, as they are usually of advanced age, debilitated, and suffering from comorbidities that either prevent successful ingestion of bowel prep or affect patients' comprehension and compliance with the regimen's instructions [1]. Suboptimal bowel preparation contributes not only to increased risk of missed pathology and patient inconvenience but also to a detrimental burden for healthcare systems, due to delayed or repeated procedures and prolonged hospital stay [2, 3]. In order to overcome these hardships, several studies have evaluated the efficacy of various interventions, i.e., different purgatives, alterations in timing of preparation administration, introduction of educational programs for physicians, nurses and patients. In this context, we conducted a systematic review of the current literature, to provide insights into types of interventions used to substantially improve inpatient's bowel preparation.

## 2. Materials and Methods

### 2.1. Protocol and Registration

This review's protocol has been registered at the International Prospective Register of Systematic Reviews (PROSPERO) under the registration number CRD42017078647.

### 2.2. Eligibility Criteria: Study Endpoint

Eligibility criteria were a priori delineated using the PICO statement as follows; P: inpatients undergoing colonoscopy for any indication; I: any type of intervention aiming to improve the quality of inpatient bowel preparation regardless of baseline disease or comorbidities; C: patients without intervention; and O: preparation's adequacy rate. Any type of trial published as full text in English language was included, while pediatric studies; meta-analyses or systematic reviews, editorials, case reports, narrative reviews, and conference abstracts; studies that did not detail patient information; and duplicate publications were excluded.

### 2.3. Information Sources and Search Strategy

A systematic computer-aided literature search of MEDLINE, Cochrane Library (Cochrane Central Register of Controlled Trials), and Google Scholar databases was performed for consistent trials. The search was initially performed on the 22^nd^ of July 2017 and repeated on the 9^th^ of June 2018—the full electronic search strategy is available in Supplementary Material A. Search was conducted independently by two investigators (PG, GT). All titles and abstracts generated from the search were screened for inclusion; further selection was conducted by obtaining full texts of identified articles to determine whether they met inclusion criteria. To fulfill a recursive search, references of all studies and reviews acquired from the electronic search were manually searched for potentially eligible studies not captured initially. Systematic reviews and meta-analyses were consulted for additional information but excluded from analysis. This systematic review was conducted according to the preferred reporting items for systematic reviews and meta-analysis (PRISMA) [4] recommendations (Supplementary Material B).

### 2.4. Study Selection

All articles retrieved from the search were screened independently by two reviewers (PG, GT). In case of uncertainty, disagreement was resolved by consensus. Titles and abstracts of all results were initially reviewed; thereafter, the full text of eligible studies was obtained and independently assessed for eligibility.

### 2.5. Data Collection Process

Data were extracted from eligible peer-reviewed articles by two investigators (PG, GT), independently using standardized extraction forms. Discrepancies regarding data extraction were also resolved by consensus.

### 2.6. Data Items

The following data were extracted from included studies: country of study origin, number of patients enrolled and their mean age, study design and setting (year, location), and number of centers. The number of patients receiving or not intervention—defined as any measure aiming to improve bowel preparation quality, including verbal or written instructions to patients, enhanced educational measures to attending healthcare professionals or other ancillary medical providers, modifications in bowel preparation regimens (qualitative and/or quantitative), and other miscellaneous measures (not classified in previous categories) before colonoscopy—was extracted. Consequently, the number of patients with adequate bowel preparation that either received or not any intervention was extracted. For the purpose of our study, bowel preparation quality was dichotomized in two groups: adequate and inadequate. Bowel preparation was considered inadequate when it scored a total of the Boston Bowel Preparation Scale (BBPS) score < 6 with at least one segment score < 2 and a total of the Ottawa Bowel Preparation Scale (OBPS) score ≥ 6, described as “poor” in the Aronchick Scale [5–7]. For studies using different bowel preparation scoring scales, results were adjusted based on authors' definitions and presented accordingly (Supplementary Material C, Table 1). Moreover, the number of patients accepting the preparation strategy, willing to repeat the procedure, reporting any adverse event (definitions according to each study are available in Supplemental Material C, Table 2); the amount of preparation received, the number of repeated colonoscopies due to inadequate bowel cleanliness; and the total length of stay were also extracted. In case of missing data, the corresponding author was contacted by email, and if no response was received, the study was excluded from the analysis.

### 2.7. Outcome Measures

Primary aim was to investigate interventions applied to inpatients undergoing colonoscopy aiming to improve colon preparation and determine their effect on the preparation's adequacy rate. Acceptance of preparation strategies, percentage of preparation received, willingness to repeat the examination, adverse events, repeat colon examinations, and duration of hospital stay comprised the secondary aims of this review.

### 2.8. Statistical Analysis

Extracted data were analyzed using the statistical software Review Manager (RevMan 5.3.5, Copenhagen, Denmark, the Nordic Cochrane Centre, the Cochrane Collaboration, 2014). Overall preparation adequacy rate and all secondary endpoints were calculated using generic inverse variance analysis, and they are presented as percentage with respective 95% confidence intervals (CI). For bowel preparation adequacy comparisons, odds ratios (ORs) and their 95% CIs were calculated. All outcomes were further compared using the random effects model (DerSimonian and Laird method). Heterogeneity among studies was measured using the *I* ^2^ with lower values representing lower levels of heterogeneity. In case of significant heterogeneity (*P* < 0.1), predefined sensitivity analysis was performed by repeating the analysis excluding one study at a time to assess potential excessive influence of a study in heterogeneity's significance. For the primary endpoint, an additional predefined sensitivity analysis according to the study design, pooling separately prospective and observational studies, was performed. Forest plots were created for visual display of results. Potential publication bias of included studies was assessed by simple inspection for symmetry of funnel plots (if included studies were less than 10) constructed by plotting the log ORs vs. precision of individual studies per outcome or by Egger's test [8] evaluated using StatsDirect 3 (StatsDirect Ltd., Sale, Cheshire, England) software, if included studies were more than ten. Finally, we used both overlapping confidence interval inspection and the test for subgroup differences provided by the statistical software in order to perform a per intervention-used subgroup analysis for each outcome.

### 2.9. Risk of Bias in Individual Studies

To assess the quality and the risk of bias of the included randomized and nonrandomized studies, we used the Cochrane collaboration tool [9] and the Newcastle-Ottawa Scale [10], respectively.

## 3. Results

### 3.1. Study Selection

**Figure 1:**
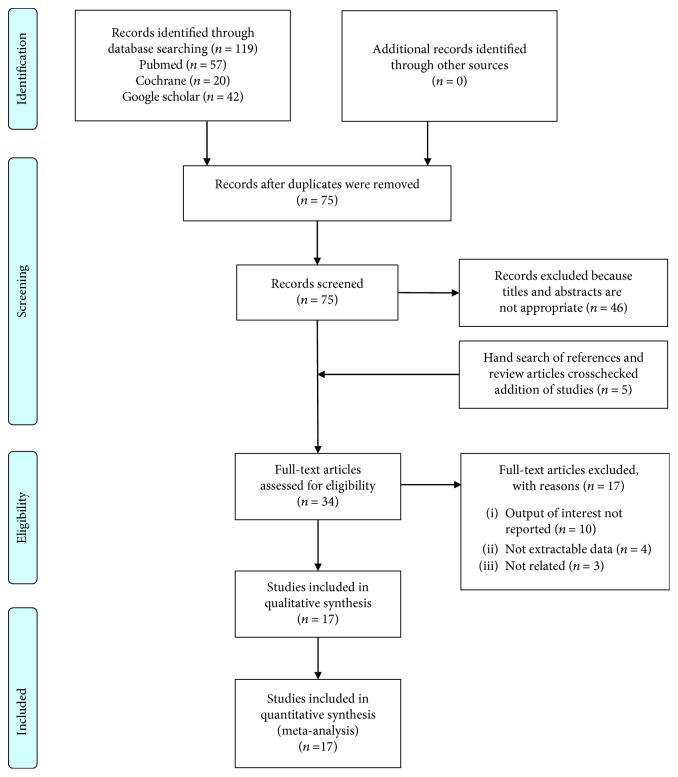
Flowchart of literature search and study selection.

The initial search generated 119 citations. After duplicate removal, 75 articles were primarily assessed by title and abstract review. Five more studies were identified through manual reference search. Finally, 34 relevant appearing results were retrieved for further assessment. Among these, 17 were excluded for various reasons, leaving 17 eligible trials to be included [11–27]. The detailed selection process is depicted on Figure 1. Eight randomized controlled trials (RCT) [15, 17, 19–21, 23, 26, 27] and nine observational cohort studies [11–14, 16, 18, 22, 24, 25] were available for analysis. In this systematic review, we classified included studies per intervention used for improving bowel cleanliness as follows: (1) education of patients and/or personnel regarding bowel preparation [11–16], (2) modification of preparation regimens [17–26], and (3) other interventions [27].

### 3.2. Characteristics of the Included Studies

Table 1 illustrates the main characteristics of the included studies, published between 2003 and 2018, enrolling 2733 inpatients. All but one [20] were monocentric; fourteen [11–13, 15–17, 19–23, 25–27] and 3 [14, 18, 24] were prospective and retrospective studies, respectively. Among the prospective studies, 8 [13, 15, 17, 19–21, 23, 26] randomized participants in 2 groups (intervention vs. control). The majority of the studies (11/17) took place in North and South America [12, 14–16, 18, 19, 21, 22, 24, 25, 27], while four studies were conducted in Europe [11, 17, 20, 26] and two in Asia [13, 23]. One retrospective case series [24] did not provide control arm and therefore was only used to measure the overall adequacy rate. A variety of different bowel preparation scales was used to evaluate cleanliness. The OBPS [6] was used in 4 [13, 21, 23, 27] and the BBPS [28] in 3 [15, 22, 26], while both the BBPS and Aronchik Scale [28, 29] were used in 2 studies [24, 25]. One study [19] used the Chilton Scale [30], while the rest (7) [11, 12, 14, 16–18, 20] used miscellaneous rating scores, consisting of 3- to 6-point scoring scales (Supplemental Material C, Table 1).

#### 3.2.1. Study Quality and Risk of Bias

**Figure 2:**
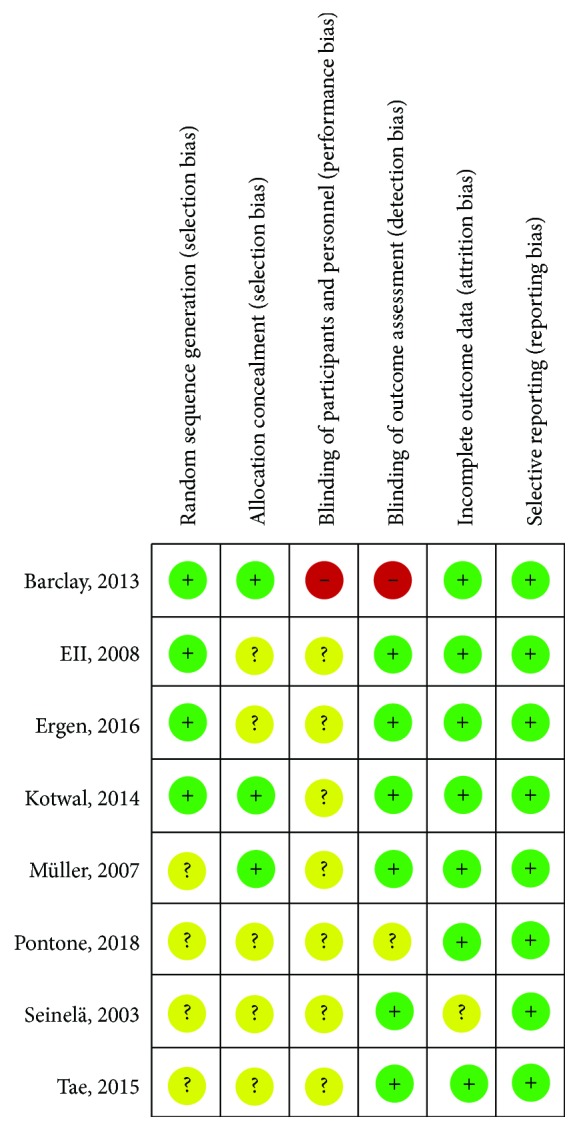
Risk of bias of included randomized controlled trials.

Figure 2 summarizes the assessment of per-study risk of bias for the randomized control studies [15, 17, 19–21, 23, 26, 27], according to Cochrane collaboration risk of bias assessment tool. Exact judgment per study and per quality domain can be found in Supplemental Material C Table 3. The overall quality of these studies is questionable—performance and detection bias being the major concerns. Risk of bias assessment for the observational studies [11–14, 16, 18, 22, 24, 25] according to Newcastle-Ottawa Scale is provided in Supplemental Material C Table 4. Four studies [12, 13, 22, 25] succeeded the highest score—receiving eight out of eight possible points—in terms of representativeness of inclusion cohorts and ascertainment of study outcomes.

#### 3.2.2. Overall Colon Cleansing Adequacy Rate

**Figure 3:**
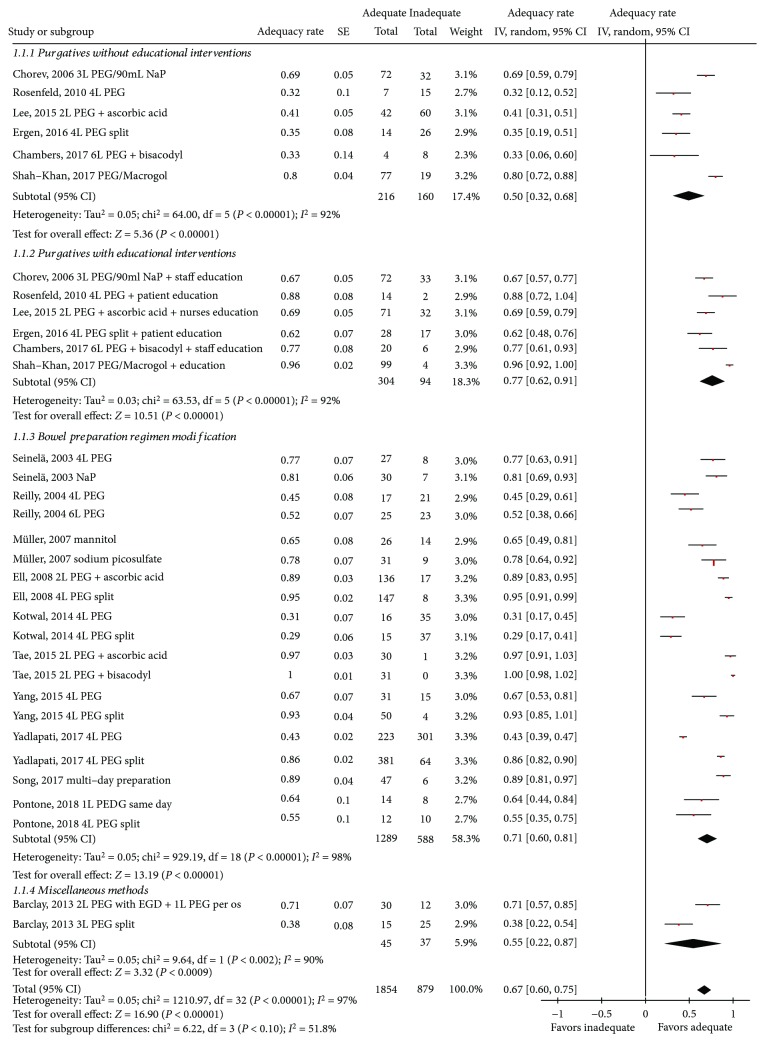
Forrest plot of studies assessing inpatients' adequacy of bowel preparation.

Overall, adequate colon cleansing was achieved in 67% (60-75%) patients (heterogeneity: *I* ^2^ = 97%, *P* < 0.001 (Figure 3)). Among the 6 studies [11–16] assessing the impact of educational interventions to either patient or physician/nurse, adequacy of bowel preparation was detected in 77% (62-91%) of the subjects in the intervention group and in 50% (32-68%) of the controls.

In the 10 studies [17–26] examining variations in bowel preparation regimens, adequate preparation was detected in 71% (60-81%) of the participants, whereas in the single study [27] examining the administration of bowel preparation through esophagogastroduodenoscopy (EGD), only 55% (22-87%) of the patients had adequate bowel preparation.

No statistically significant difference among the three groups of interventions was found (Figure 3). Finally, there was no evidence of publication bias (Egger's test: 1.64 (95% CI = −1.37 to 4.66), *P* = 0.26; Supplemental Material D Figure 1A).

#### 3.2.3. Educational Interventions

There was one RCT [15] and 5 nonrandomized prospective studies [11–14, 16] evaluating the effect of educational interventions on bowel preparation. A detailed description about each study's educational intervention is presented in Supplemental Material C Table 5. In two studies, investigators assigned participants to the intervention arms to receive either an educational booklet on colonoscopy preparation [15] or an extra brief counselling and written instructions regarding the methods and rationale of bowel preparation [12], respectively. Three studies [11, 13, 14] educated the personnel involved in patients' preparation. Special leaflets, lectures, and/or presentations were used to educate nurses, who guided study participants before and during bowel preparation, and consequent comparison with either the preintervention period [11, 14] or wards where noneducated nurses participated [13] was made. Finally, one study [16] is aimed at educating both personnel and patients.

**Figure 4:**
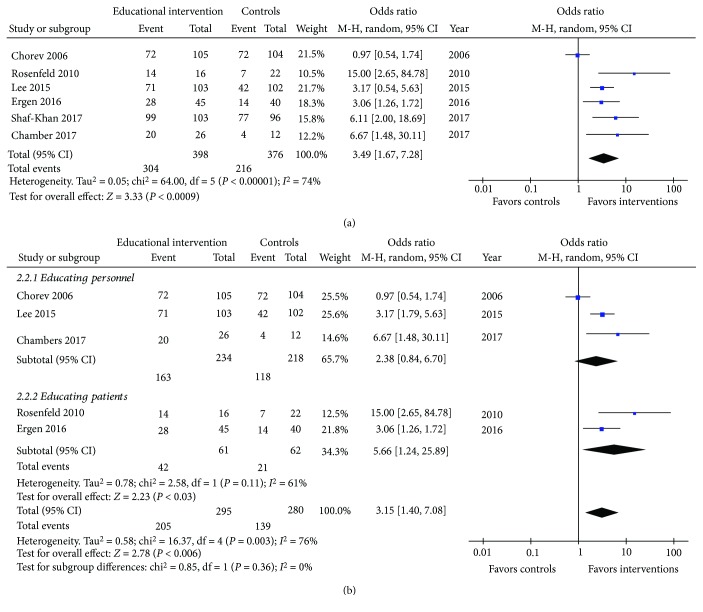
Forrest plot of studies assessing the effect of educational intervention on bowel preparation quality of (a) overall and (b) per targeted population.

Overall, 774 patients were included in the aforementioned studies [11–16]. Of them, 304/398 and 216/376 achieved adequate bowel preparation in the intervention group and the control group, respectively (OR (95% CI): 3.49 (1.67-7.28), *P* = 0.0009; *I* ^2^ = 74%, *P* = 0.002; Figure 4(a)). No publication bias was detected (Supplemental Material D Figure 1B. During the step-by-step sensitivity analysis, one study [11] was identified to be responsible for the significant heterogeneity. However, its exclusion did not alter the meta-analytic outcome (OR (95% CI): 4.04 (2.62-6.25), *P* < 0.00001; *I* ^2^ = 5%, *P* = 0.38). Furthermore, we tested whether educating either personnel or patients themselves had a different effect on preparation's adequacy. In this subgroup analysis (Figure 4(b)), no significant difference between the two groups (test for subgroup differences: chi^2^ = 0.85, df = 1, *P* = 0.36) was detected. Removing the study by Chorev et al. [11] eliminated the detected heterogeneity without altering the outcome (OR (95% CI): 3.48 (2.04-5.96), *P* < 0.00001; *I* ^2^ = 0%, *P* = 0.37 and 5.66 (1.24-25.89), *P* = 0.03; *I* ^2^ = 61%, *P* = 0.11 for educating personnel and patients, respectively). Since the study by Shah-Khan et al. [16] evaluated the education of both personnel and patients, it was excluded from the aforementioned subgroup analysis. Finally, the presence of a sole RCT prevented a subgroup analysis according to the study design.

#### 3.2.4. Various Preparation Regimens

Ten studies including 1802 individuals examined the impact of cathartics and alterations in timing of their administration on bowel cleansing [17–26]. Heterogeneity of regimens precluded meta-analysis; thus, they are presented in a qualitative-narrative manner organized in 3 subgroups: (a) various purgatives, (b) combinations of low-volume preparations with adjunctive agents, and (c) effect of timing of preparation administration (split dose vs. single dose). Reilly and Walker [18] deemed 6-L PEG (polyethylene glycol) with an additional second preparation (e.g., laxatives, tap water enemas, and Fleet enemas) as the optimal strategy while Seinelä et al. [17] demonstrated no significant benefit of sodium phosphate over PEG regarding adequacy of cleansing (81% vs. 77%, *P* = not available). Müller et al. [19] randomized subjects to receive mannitol or sodium picosulfate reporting equivalent results between groups. Moreover, in a large (*n* = 308) German multicenter noninferiority RCT, 2 L of PEG plus ascorbic acid achieved similar colon cleanliness compared to 4 L PEG solution (88.9% vs. 94.8%, –5.9% with a lower limit of the 1-sided 97.5% confidence interval –12.0%, within the limits for noninferiority set before the study) [20]. Similarly, in a single-center randomized pilot study evaluating the impact of a same-day, 1-liter PEG on the diagnostic rating and tolerability, no difference compared to split-dose 4 L PEG was noted (63% vs. 56%, *P* = 0.64). Thus, same-day, 1 L-PEG bowel preparation could be introduced for selected inpatients [26]. In another randomized Korean study, the efficacy of low-volume (2–L) PEG with ascorbic acid was comparable to that of 2 L of PEG plus bisacodyl [23]. Regarding timing of bowel preparation, split-dose PEG preparation has been reported to be superior to same-day preparation [22]. On the contrary, a randomized, single-center study reported no difference between split-dose and morning-only PEG preparations (mean total Ottawa Scale score: 7.38 ± 3.65 vs. 7.15 ± 3.58, *P* = 0.75) [21]. Yadlapati et al. [25] reported a higher rate of adequate bowel preparation in patients receiving 4 L PEG as split dose compared to same-day regimen (85.7% vs. 42.5%, *P* < 0.01). Finally, implementation of a multiday bowel preparation regimen in 53 spinal cord injury patients led to adequate bowel cleanliness in 89% of the participants [24]. The cleansing rate was not affected by the study design (73% (63-83%) vs. 65% (49-81%) for RCTs and observational studies, respectively; test for subgroup differences (chi^2^ = 0.63, df = 1, *P* = 0.43).

#### 3.2.5. Miscellaneous Methods

In a single-center study, Barclay [27] administered 2 L of PEG in 42 inpatients through a gastroscope right after diagnostic EGD. Colonoscopy took place the following day, after oral ingestion of one more liter of PEG. The control group consisted of 40 patients undergoing colonoscopy prepared with 3 L of PEG solution orally. Using OBPS, EGD-assisted PEG administration was associated with better quality of bowel preparation (4.1 ± 2.8 vs. 6.5 ± 3.1; *P* < 0.0005).

#### 3.2.6. Secondary Endpoints

Table 2 summarizes data on the secondary endpoints. In terms of acceptance of preparation strategies, 9 studies with 17 sets of data were identified [13, 17, 19–21, 23, 24, 26, 27]. Overall, 72% (64-80%) of the participants accepted the administered preparation. In the single study [13] examining purgatives with and without educational interventions, the preparation acceptance rate was higher among patients allocated to receive education compared to those without (92% (86-98%) vs. 61% (51-71%), respectively). In the subgroup of 7 studies [17, 19–21, 23, 24, 26] (13 sets of data) assessing modifications of different preparation regimens, the overall acceptance rate was 73% (64-82%). Finally, in the two-arm study [27], where either EGD-assisted or conventional per os preparation was administered, the acceptance rate of the intervention was 74% (60-88%) compared to 45% (29-61%) of the control arm (Supplemental Material D, Figure 2A).

Eight studies (16 sets of data) [13, 14, 17, 20–23, 27] reported on the adequacy of the amount of bowel preparation received, as defined per study (50% to 100% of the volume of the preparation). The majority (91% (88-95%)) of included patients received an adequate amount of preparation. Among the 5 studies [17, 20–23] examining the effect of various purgative regimens on bowel preparation, 91% (87-94%) of patients succeeded in receiving adequate volume of preparation. In two studies [13, 14], 89% (83-95%) of participants in the arm receiving purgatives without educational interventions consumed an adequate amount of the provided preparation, compared with 98% (96-100%) of the participants in the educational intervention arms. Finally, in the study of Barclay [27], 93% (85-100%) of the patients on the EGD-assisted arm and 85% (73-97%) of those on 3 L of PEG split-dose per os arm received an adequate amount of preparation, as defined by the author (Supplemental Material D, Figure 2B).

Seven studies with 13 sets of data [13, 17, 19, 21–23, 31] indicated that 77% (69-85%) of the participants were willing to undertake the same bowel preparation, if needed. In the study by Lee et al. [13], participants in both arms—with and without educational intervention—showed similar rates of willingness to repeat the examination (74% (66-82%) and 84% (76-92%), respectively); all patients prepared with 2 liters of PEG plus ascorbic acid. In the subgroup of 5 studies [17, 19, 21–23] evaluating different bowel preparation regimens, the willingness to repeat rate was 79% (69-90%) (Supplemental Material D, Figure 2C).

Nine studies [13, 17, 19–24, 26] with 17 sets of data reported adverse events in 40% (37-42%) of the patients. Reported adverse events are listed in Supplemental Material C Table 2. There were no serious adverse events or preparation termination inducing adverse events. The adverse event rate was similar among the various subgroups. In the study of Lee et al. [13], 47% (37-57%) of the participants in the arm without educational intervention reported at least one adverse event compared to 35% (25-45%) of the participants in the arm with nurses' education. In the subgroup of studies [17, 19–24, 26] with various modified bowel preparation regimens, the adverse event rate was 39% (36-42%) (Supplemental Material D, Figure 2D).

Three studies [11, 15, 25] presented results regarding the length of stay and repeated endoscopies (Table 2). Ergen et al. [15] reported that mean total hospital stay was 6 days in patients receiving 4 L PEG split-dose plus education vs. 5 days in those receiving the same regimen without education; Yadlapati et al. [25] reported 8 days mean hospital stay after preparation commencement for patients receiving same-day 4 L PEG compared to 6.9 days for those getting 4 L split-dose PEG. Two studies [11, 15] reported on the need to repeat colonoscopies because of inadequate bowel preparation. No difference was noted between patients receiving purgatives with personnel education and those only on purgatives (OR (95% CI): 0.93 (0.47-1.81); *P* = 0.82, *I* ^2^ = 0%, *P* = 0.47).

## 4. Discussion

A number of inpatient-related factors may contribute to inadequate bowel cleansing [2]. In the absence of guidelines or recommendations, several methods have been implemented to improve bowel preparation scores [13, 15, 19–21, 26]. Despite their application, the overall adequacy rate of bowel preparation remains low. Our systematic review and meta-analysis confirm the low preparation adequacy rate (67%) among inpatients undergoing colonoscopy.

Our analysis showed that educating either the patients or the hospital personnel or both may pose certain effect on inpatients' bowel preparation quality. Educational interventions (paper-based interventions, videos, reeducation phone calls the day before colonoscopy, or in-person education by physicians) have been established from outpatients' studies as efficient methods to optimize colon preparation outcome [32]. However, the evidence to strongly support a similar conclusion regarding inpatients is quite low deriving only from 1 RCT and 5 nonrandomized studies. Specifically designed booklets [15] and written instructions [12] have been used to assist inpatients not only to discern the importance of adequate bowel preparation but also to increase their compliance by clarifying potential queries related to the procedure (adverse events, time points of regimen administration, etc.). Training healthcare professionals by using lectures and presentations [11, 13, 14] about the importance of adequate preparation and how to achieve it and recording adherence to the preparation plan through electronic documentation [11, 14] might also enhance the effectiveness of provided instructions and decrease the rate of inadequate preparations. Still, all the abovementioned interventions are far from being perfect, as the overall colon cleansing adequacy rate remains suboptimal.

In addition, our analysis did not find solid evidence to support that specific types of cathartics or alterations in timing of their administration could result in better mucosa visualization. Although several approaches are available, the ideal bowel preparation regimen for inpatients remains to be determined, yet. Given the fact that several predictors of inadequate preparation are to be anticipated (e.g., advanced age, deteriorated health status, multiple medications, and comorbidities), this might be a particularly difficult task [2]. PEG-based regimens could be considered as the first step in any preparation strategy as they are more likely to achieve adequate bowel cleanliness retaining at the same time excellent patient safety profile. However, even they are not the optimal choice as their efficacy may be severely hampered by poor tolerability and compliance due to inability to drink 4-L PEG formulations, unpleasant taste, lack of comprehension, and complexity of the preparation instructions. Thus, “hybrid” bowel preparations, i.e., low-dose PEG with adjunctive agents like ascorbic acid that display equal effectiveness to the standard 4 L regimen could represent a useful alternative [20, 33].

Since no single intervention has been shown to be efficacious in reaching the optimal level of bowel preparation in inpatients, one could speculate that multiple, combined strategies based on a case by case decision may have the potential to influence the final outcome. Indeed, this is the key message of a recent trial, where implementation of a standardized order set with split-dosing regimen, provision of written educational material to patient, and active nursing facilitation to the process overall resulted in significant positive improvements in the rate of acceptable inpatient bowel preparation [34].

Core strengths of the meta-analysis are the comprehensive and contemporaneous search strategy, including a recursive search of the literature of selected articles. To the best of our knowledge, this is the first study systematically addressing all available interventions to improve bowel preparation in inpatients.

We acknowledge a series of limitations in our study. The principal limitation lies in the heterogeneity encountered, calling for careful interpretation of our results. The latter mainly arises from the characteristics of the meta-analyzed evidence: retrospective, single-center setting, inadequate statistical power, small samples, and combination of randomized and observational studies, arbitrary classification of the reviewed interventions, and bowel preparation scales used. In an effort to explore the evident heterogeneity, we performed predefined sensitivity analyses; nevertheless, ecological bias cannot be excluded. Even the existing evidence supporting that educational interventions reduce the rate of inadequate colon cleansing could be of higher quality. One could argue that the presence of significant heterogeneity and questionable—in some instances—study's quality included may challenge the validity of our results; however, our review enhances existing literature by specifically highlighting the potential role of educational interventions in inpatients bowel preparation adequacy and how current studies may still offer guidance in everyday clinical practice. Moreover, information regarding the exact stationary status of inpatients was absent, while concomitant medications were not systematically analyzed. Finally, local factors (e.g., staff availability) that might affect each intervention's efficacy remain underrated.

In conclusion, this study highlights the inadequate level of bowel preparation in inpatients undergoing colonoscopy, although several interventions have been implemented to increase it. However, educational interventions provided to patients and health care personnel reduce the rate of inadequate colon cleansing.

## Figures and Tables

**Table 1:** Summary of included studies.

Author, year	Country	Study design	Patients enrolled, *n*	Age (mean), intervention vs. no intervention	Intervention	Type of preparation regimen used (only for studies evaluating educational interventions)	Scale assessing bowel preparation quality^†^	Patients achieving adequate preparation in the intervention group, *n* ^††^/patients enrolled in this arm, *n*	Patients achieving adequate preparation without intervention, *n*/patients enrolled in this arm, *n*
*Educational interventions*									
Chorev, 2006*ˠ*	Israel	Prospective observational, single center	209	68.5	Physician and nurse educational program (lectures and instruction on preparation); oral, written instructions provided to all patients	Pts in both cohorts received >75 years or with moderate to severe heart or kidney failure were given PEG, 3 L, the evening before. All others were given sodium phosphate, 2 bottles of 45 mL each, to be taken with 12 glasses of tap water. Time elapsing between last sip of purgative and colonoscopy is NA	Adapted quality rating scale	72/105	72/104
Rosenfeld, 2010	Canada	Prospective observational, endoscopist blinded, single center (first 8 weeks assigned to intervention, the following 8 weeks to conventional)	38	65.1 vs. 67.9	Patient education (instruction group provided with 5 min verbal and written instructions prior to colonoscopy vs. no instruction)	Pts in both cohorts received 4 L of PEG bowel preparation with a clear liquid diet on the day before colonoscopy. Time elapsing between last sip of purgative and colonoscopy is NA	Adapted quality rating scale	14/16	7/22
Lee, 2015^∗^	South Korea	Prospective, double blind nonrandomized controlled, single center	205	64 vs. 63	Education for ward nurses (educational leaflet and lecture vs. no education)	Pts received low-residue diet 2 days before colonoscopy; on the day before colonoscopy, pts were provided a soft diet for dinner before 6 pm and, after that time, only clear water. 2 L of PEG plus ascorbic acid was ingested—250 mL every 10 minutes. For colonoscopies performed in the morning, a split-dose bowel preparation (half-dose of purgative at 8 : 00 pm on the day before the procedure and the remaining 1 L on the morning of the day of the procedure). For afternoon colonoscopies, a full dose (2 l) of PEG plu Asc between 6 : 00 and 8 : 00 am on the day of the procedure. All colonoscopies were performed between 2 and 8 hours after the purgative intake was complete	OBPS	71/103	42/102
Chambers, 2016	USA	Retrospective, single center	38	NA	Patient and nurse education (preprocedure education)	All patients received half of the 6 L preparation and a bisacodyl pill	Adapted quality rating scale	20/26	4/12
Ergen, 2016	USA	Prospective, randomized, single blind, controlled trial, single center	85	57 vs. 58	Patients given an educational booklet before colonoscopy	All pts received a standard preparation: clear liquid diet the day prior to the day of the procedure followed by split-dose PEG. Patients are instructed to consume 2 L between 6 pm and 8 pm the night prior to colonoscopy and 2 L between 5 am and 7 am on the day of colonoscopy	BBPS	28/45	14/40
Shah-Khan, 2017	USA	Prospective nonrandomized, single center	199	NR	Multiphase intervention program involving physicians and nursing staff education, implementation of electronic order set, and patient education	NA	Adapted quality rating scale	99/103	77/96

*Bowel preparation regimens*									
Seinelä, 2003^∗∗^	Finland	Prospective, randomized, endoscopist blinded, single center	72	84	NaP vs. 4 lit PEG standard dosing		Adapted quality rating scale	30/37	27/35
Reilly, 2004	USA	Retrospective, cohort, single center	101	NA	4 lit PEG vs. 6 lit PEG		Adapted quality rating scale	17/38	25/48
Müller, 2007	Brazil	Prospective, randomized, single center	80	62.4 vs. 60.6	Mannitol-based preparation regimen vs. sodium picosulfate-based regimen		Chilton Scale	26/40	31/40
Ell, 2008^∗∗^	Germany	Prospective, randomized, single blinded, multicenter	308	58 vs. 59.6	2 lit PEG plus ascorbic vs. 4 lit PEG solution		Adapted quality rating scale	136/153	147/155
Kotwal, 2014	USA	Prospective, randomized, endoscopist blinded, single center	103	52.8 vs. 57.4	Morning only preparation (4 lit PEG between 5-9 am on the day of colonoscopy vs. split-dose PEG 2 lit - 2 lit (noninferiority study)		OBPS	16/51	15/52
Yang, 2015	USA	Prospective observational, multiphase, single center	100	63.2 vs. 63.7	Nurse education and electronic order set and split-dose preparation vs. standard full-dose 4 lit PEG		BBPS	50/54	31/46
Tae, 2015	Korea	Prospective, randomized, controlled, single center	62	56.8 vs. 52.4	Low-volume 2 lit PEG containing ascorbic vs. 2 lit PEG plus 20 mg bisacodyl		OBPS	30/31	31/31
Song, 2017^∗∗∗^	USA	Retrospective, case series	53	64.1	Multiday preparation regimen		BBPS or Aronchick Scale^§^	47/53	NA
Yadlapati, 2017	USA	Pragmatic, two-cohort-quasi-experimental study; postintervention cohort prospectively built; prep-intervention cohort: historic data	879	58.2 vs. 57.1	Implementation of split-dose PEG bowel preparation algorithm combined with an electronic dataset vs. single-dose 4 L PEG solution the evening before inpatient colonoscopy		BBPS or Aronchick Scale^§^	381/445	223/534
Pontone, 2018	Italy	Prospective, randomized, controlled single-center, pilot study	44	64 vs. 63	Same-day 1 L PEG bowel preparation on the morning of the colonoscopy vs. split-dose 4 L PEG (3 L the evening before and 1 L in the morning of the day of colonoscopy)		BBPS	14/22	12/22

*Miscellaneous methods*									
Barclay, 2013^∗^	USA	Prospective, randomized, controlled, single center	82	73 vs. 73.5	EGD-assisted bowel prep (2 lit PEG administered endoscopically into distal duodenum plus 1 L PEG orally the following day) vs. split-dose PEG preparation (2 lit PEG orally the evening prior and 1 lit PEG orally the following day)		OBPS	30/42	15/40

NA: not applicable; BBPS: Boston Bowel Preparation Scale; OBPS: Ottawa Bowel Preparation Scale; PEG: polyethylene glycol; NaP: sodium phosphate; EGD: esophagogastroduodenoscopy; ^†^as evaluated in each study; detailed information regarding quality preparation assessment scale of each study is available in Supplemental Material C Table 1; ^††^adequacy of bowel preparation was defined according to each study's criterion; *ˠ*the value of stuff educational program on the preparation of hospitalized patients was examined as secondary endpoint; ^∗^inadequate preparation was defined as an overall Ottawa score ≥ 6; lower score indicates better bowel cleansing; ^∗∗^study evaluating different bowel preparation regimens in inpatients; ^∗∗∗^study evaluating efficacy of a multiday colonoscopy bowel preparation; ^§^studies using two scales to assess bowel preparation.

**Table 2:** Secondary endpoints.

	Author, year	Acceptance of preparation strategies (*n*/*N*)	Patients receiving adequate preparation (*n*/*N*)	Willingness to repeat colonoscopy (*n*/*N*)	AE (*n*/*N*)	Hospital stay (days)	Repeat colon examinations (*n*/*N*)
Educational interventions	Chorev, 2006	NR	177/209 (overall; not per intervention)	NR	NR	NR	20/105
							20/104
	Rosenfeld, 2010	NR	NR	NR	NR	NR	NR
	Lee, 2015	95/103	101/103^¶^	86/103	36/103	NR	NR
		62/102	91/102^¶^	75/102	48/102		
	Ergen, 2016	NR	NR	NR	NR	6	0/45
						5	1/40
	Chambers, 2017	NR	26/26^¶¶^	NR	NR	NR	NR
			12/12^¶¶^				
	Shah-Khan, 2017	NR	NR	NR	NR	NR	NR

Bowel regimens modification	Seinela, 2003	26/35	31/35^¶¶¶¶^	13/35	7/35^∗^	NR	NR
		23/37	36/37^¶¶¶¶^	18/37	20/37^∗^		
	Reilly, 2004	NR	NR	NR	NR	NR	NR
	Muller, 2007	32/40	NR	32/40	6/40	NR	NR
		37/40		37/40	10/40		
	Ell, 2008	113/153	130/153	NR	73/153	NR	NR
		82/155	134/155		86/155		
	Kotwal, 2014	33/51	43/51	36/51	36/51	NR	NR
		38/52	48/52	46/52	28/52		
	Yang, 2015	NR	37/46^¶¶¶^	NR	26/46	NR	NR
			52/54^¶¶¶^	49/54	19/54		
	Tae, 2015	24/31	30/31^¶¶¶^	29/31	14/31	NR	NR
		18/31	29/31	30/31	14/31		
	Song, 2017^¥^	50/53	NR	NR	5/53	NR	NR
	Yadlapati, 2017	NR	NR	NR	NR	8 ± 11.4	24/524
						6.9 ± 8.8	9/445
	Pontone, 2018	15/22	NR	NR	8/22	3	NR
		16/22			6/22	6	

Others	Barclay, 2013	31/42	39/42	NR	NR	NR	NR
		18/40	34/40	NR	NR	NR	NR

^∗^refers to nausea that statistically differed between the two groups; ^¶^at least 80% of preparation; ^¶¶^at least 50% of preparation; ^¶¶¶^at least 75% of preparation; ^¶¶¶¶^100% of preparation received; ^¥^study evaluating efficacy of a multiday colonoscopy bowel preparation; all enrolled patients received the same intervention.

## Supplementary Materials

Supplementary Materials Supplemental Material A: search strategy. Supplemental Material B: preferred reporting items for systematic reviews and meta-analyses (PRISMA) 2009 checklist. Supplemental Material C Table 1: bowel preparation rating scales used in the included studies. Supplemental Material C Table 2: assessment of secondary endpoints. Supplemental Material C Table 3: risk of bias assessment of included randomized studies and authors' judgement. Supplemental Material C Table 4: risk of bias assessment of included observational studies using the Newcastle-Ottawa Scale. Supplemental Material C Table 5: educational interventions described in the included studies. Supplemental Material D Figure 1: funnel plot of studies included in the meta-analysis assessing (A) the overall adequacy rate and (B) the impact of educational intervention to improve inpatient bowel preparation quality. Supplemental Material D Figure 2: forest plots of studies included in the meta-analysis assessing acceptance of preparation strategies (A), amount of preparation received (B), willingness to repeat the examination (C), and adverse events (D).
