# Supplementary material for: Strategies to Improve Inpatients' Quality of Bowel Preparation for Colonoscopy: A Systematic Review and Meta-Analysis
Source: Gastroenterol Res Pract. 2019 May 2;2019:5147208. doi: 10.1155/2019/5147208 (PMC6525904; doi:10.1155/2019/5147208)
Supplement: Supplementary Materials — Supplemental Material A: search strategy. Supplemental Material B: preferred reporting items for systematic reviews and meta-analyses (PRISMA) 2009 checklist. Supplemental Material C Table 1: bowel preparation rating scales used in the included studies. Supplemental Material C Table 2: assessment of secondary endpoints. Supplemental Material C Table 3: risk of bias assessment of included randomized studies and authors' judgement. Supplemental Material C Table 4: risk of bias assessment of included observational studies using the Newcastle-Ottawa Scale. Supplemental Material C Table 5: educational interventions described in the included studies. Supplemental Material D Figure 1: funnel plot of studies included in the meta-analysis assessing (A) the overall adequacy rate and (B) the impact of educational intervention to improve inpatient bowel preparation quality. Supplemental Material D Figure 2: forest plots of studies included in the meta-analysis assessing acceptance of preparation strategies (A), amount of preparation received (B), willingness to repeat the examination (C), and adverse events (D). [file 5147208.f1.docx]

**Supplemental Material A.** Search Strategy

| **PubMed (Date Run: 09/06/2018)** | | | |
| --- | --- | --- | --- |
| **Step** | **Search strategy** | **Found** | **Time** |
| **#1** | Search ((((((((((((((inpatients) OR Inpatient | 99600 | 11:48:41 |
| **#2** | Search (((((((((((((Colonoscopy) OR Colonoscopies) OR Colonoscopic Surgical Procedures) OR Colonoscopic Surgical Procedure) OR Procedure, Colonoscopic Surgical) OR Procedures, Colonoscopic Surgical) OR Surgical Procedure, Colonoscopic) OR Surgery, Colonoscopic) OR Surgical Procedures, Colonoscopic) OR Colonoscopic Surgery) OR Colonoscopic Surgeries) OR Surgeries, Colonoscopic | 37585 | 11:49:23 |
| **#3** | Search (((((((((((((Bowel preparation) OR Bowel Evacuants  Evacuants) OR Bowel Purgatives) OR Bowel Preparation Solutions) OR Preparation Solutions, Bowel) OR Solutions, Bowel Preparation | 304638 | 11:50:09 |
| **#4** | #1 AND #2 AND #3 | 57 | 11:52:51 |

| **Cochrane Library (Date Run: 22/07/2017)** | | |
| --- | --- | --- |
| **Step** | **Search strategy** | **Found** |
| **#1** | Inpatient or Inpatient | 8607 |
| **#2** | Colonoscopy or Colonoscopy, Virtual or Virtual Colonoscopy | 3701 |
| **#3** | Bowel preparation or Bowel Evacuants or Evacuants, Bowel or Bowel Preparation Solutions or Preparation Solutions, Bowel or Solutions, Bowel Preparation | 1798 |
| **#4** | #1 and #2 and #3 | 20 |

| **Google Scholar (Date Run: 22/07/2017)** | | |
| --- | --- | --- |
| **Step** | **Search strategy** | **Found** |
|  | Inpatient bowel OR preparation "colonoscopy" | 42 |

**Supplemental Material B.** Preferred Reporting Items for Systematic Reviews and Meta-Analyses checklist

| **Section/topic** | **#** | **Checklist item** | **Reported on page #** |
| --- | --- | --- | --- |
| **TITLE** | | |  |
| Title | 1 | Identify the report as a systematic review, meta-analysis, or both. | 1 |
| **ABSTRACT** | | |  |
| Structured summary | 2 | Provide a structured summary including, as applicable: background; objectives; data sources; study eligibility criteria, participants, and interventions; study appraisal and synthesis methods; results; limitations; conclusions and implications of key findings; systematic review registration number. | 2 |
| **INTRODUCTION** | | |  |
| Rationale | 3 | Describe the rationale for the review in the context of what is already known. | 4 |
| Objectives | 4 | Provide an explicit statement of questions being addressed with reference to participants, interventions, comparisons, outcomes, and study design (PICOS). | 4 |
| **METHODS** | | |  |
| Protocol and registration | 5 | Indicate if a review protocol exists, if and where it can be accessed (e.g., Web address), and, if available, provide registration information including registration number. | 5 |
| Eligibility criteria | 6 | Specify study characteristics (e.g., PICOS, length of follow-up) and report characteristics (e.g., years considered, language, publication status) used as criteria for eligibility, giving rationale. | 5 |
| Information sources | 7 | Describe all information sources (e.g., databases with dates of coverage, contact with study authors to identify additional studies) in the search and date last searched. | 5 |
| Search | 8 | Present full electronic search strategy for at least one database, including any limits used, such that it could be repeated. | 5-6  Appendix A |
| Study selection | 9 | State the process for selecting studies (i.e., screening, eligibility, included in systematic review, and, if applicable, included in the meta-analysis). | 6 |
| Data collection process | 10 | Describe method of data extraction from reports (e.g., piloted forms, independently, in duplicate) and any processes for obtaining and confirming data from investigators. | 6 |
| Data items | 11 | List and define all variables for which data were sought (e.g., PICOS, funding sources) and any assumptions and simplifications made. | 6-7 |
| Risk of bias in individual studies | 12 | Describe methods used for assessing risk of bias of individual studies (including specification of whether this was done at the study or outcome level), and how this information is to be used in any data synthesis. | 9 |
| Summary measures | 13 | State the principal summary measures (e.g., risk ratio, difference in means). | 7 |
| Synthesis of results | 14 | Describe the methods of handling data and combining results of studies, if done, including measures of consistency (e.g., I^2^) for each meta-analysis. | 8 |

|  |  |  |  |
| --- | --- | --- | --- |

| **Section/topic** | **#** | **Checklist item** | **Reported on page #** |
| --- | --- | --- | --- |
| Risk of bias across studies | 15 | Specify any assessment of risk of bias that may affect the cumulative evidence (e.g., publication bias, selective reporting within studies). | 9 |
| Additional analyses | 16 | Describe methods of additional analyses (e.g., sensitivity or subgroup analyses, meta-regression), if done, indicating which were pre-specified. | 9 |
| **RESULTS** | | |  |
| Study selection | 17 | Give numbers of studies screened, assessed for eligibility, and included in the review, with reasons for exclusions at each stage, ideally with a flow diagram. | 10 |
| Study characteristics | 18 | For each study, present characteristics for which data were extracted (e.g., study size, PICOS, follow-up period) and provide the citations. | 10, Table 1 |
| Risk of bias within studies | 19 | Present data on risk of bias of each study and, if available, any outcome level assessment (see item 12). | 11 |
| Results of individual studies | 20 | For all outcomes considered (benefits or harms), present, for each study: (a) simple summary data for each intervention group (b) effect estimates and confidence intervals, ideally with a forest plot. | 11-12 |
| Synthesis of results | 21 | Present results of each meta-analysis done, including confidence intervals and measures of consistency. | 12-17 |
| Risk of bias across studies | 22 | Present results of any assessment of risk of bias across studies (see Item 15). | 12, Figure 2, Appendix C Supp. Table 3 |
| Additional analysis | 23 | Give results of additional analyses, if done (e.g., sensitivity or subgroup analyses, meta-regression [see Item 16]). | 14 |
| **DISCUSSION** | | |  |
| Summary of evidence | 24 | Summarize the main findings including the strength of evidence for each main outcome; consider their relevance to key groups (e.g., healthcare providers, users, and policy makers). | 18-21 |
| Limitations | 25 | Discuss limitations at study and outcome level (e.g., risk of bias), and at review-level (e.g., incomplete retrieval of identified research, reporting bias). | 20 |
| Conclusions | 26 | Provide a general interpretation of the results in the context of other evidence, and implications for future research. | 20-21 |
| **FUNDING** | | |  |
| Funding | 27 | Describe sources of funding for the systematic review and other support (e.g., supply of data); role of funders for the systematic review. | 1 |

**Supplemental Material C. Tables**

| **Supplemental Material C Table 1.** Βowel preparation rating scales used in included studies | | | |
| --- | --- | --- | --- |
| **Scale used** | **Scale description** | **Adequacy definition** | **References** |
| Boston Bowel Preparation Scale (BBPS) | Four-point scoring applied to each of the three colon segments: right colon (cecum, ascending), transverse (including hepatic, splenic flexures), and left colon (descending colon, sigmoid and rectum). 0 = Unprepared colon segment with mucosa not seen due to solid stool that cannot be cleared; 1 = Portion of mucosa of the colon segment seen, but other areas of the colon segment not well seen due to staining, residual stool and/or opaque liquid; 2 = Minor amount of residual staining, small fragments of stool and/or opaque liquid, but mucosa of colon segment seen well; 3 = Entire mucosa of colon segment seen well with no residual staining, small fragments of stool or opaque liquid. Each region receives from 0 to 3 and these segment scores are summed for a total score from 0 to 9 | Total BBPS score ≥6 and all segment scores ≥2 | Ergen, 2016; Yang, 2015; Song, 2017; Yadlapati, 2017; Pontone 2018 |
| Ottawa Bowel Preparation Scale (OBPS) | An assessment was performed on the overall fluid volume (0=small, 1=moderate, and 2=large) of the entire colon and its cleanliness rated from 0 to 4 (*0=excellent, 1=good, 2=fair, 3=poor, and 4=inadequate*), for the right colon, mid-colon, and rectosigmoid colon. The total score was calculated as sum of the overall fluid volume and each of the segmental cleanliness scores and ranged from 0 to 14; a higher score indicates poorer preparation | Ottawa score <6 | Lee, 2015; Kotwal, 2014; Tae, 2015; Barclay, 2013 |
| Aronchick scale | Scoring before attempt of washing or suctioning: *1* (excellent): small volume of clear liquid or >95% of the surface seen*; 2* (good): large volume of clear liquid covering 5–25% of the surface but >90% seen; *3 (fair):* some semi-solid stool that could be suctioned or washed away but>90% of surface seen; *4 (poor):* semi-solid stool not suctioned or washed away and <90% of surface seen; *5 (inadequate):* repeat preparation | “Excellent”, “good” and “fair” preparations | Song, 2017; Yadlapati, 2017 |
| Chilton Scale | *Poor:* solid residues beyond the cecum and ascending colon; *intermediate*: large quantity of liquid residue and/or small quantity of solid residues limited to cecum and ascending colon; *good*: colon partially clean with moderate quantity of liquid residue cleaned by aspiration; *excellent*: clean colon with very little liquid residue | “Excellent” and “good” preparations | Muller, 2007 |
| Various adapted quality rating scales | *Good* preparation: no or minimal solid stool with some amounts of clear fluid requiring suctioning; *Fair*: collections of semisolid debris that are cleared with difficulty; *Poor*: solid or semisolid debris that cannot be effectively cleared | “Good” and “Fair” preparations | Chorev, 2006 |
|  | The overall quality of bowel preparation was rated with a scoring system ranging from 0 to 4; no solid fees (score=0), presence of solid feces with less than 90% of the colonic mucosa visible (score =4). | Score from 0 to 2 | Rosenfeld, 2010 |
|  | Adequate, inadequate or poor according to endoscopist | “Adequate” preparation | Shah-Khan, 2017 |
|  | Grades: *1*= excellent (small volume of clear liquid), *2*= good (large volume of clear fluid), *3*= fair (moderate amounts of stool that could be suctioned away), *4*= poor (large amounts of stool that could not be suctioned away). | “Excellent” or “good” preparation | Seinela, 2003 |
|  | Excellent, very good, good, adequate, fair, inadequate or poor according to endoscopist | “Excellent”, “very good”, “good” and “adequate” | Reilly, 2004 |
|  | Scoring system ranging from A to D; *A)* all bowel segments clean (score of 3 or 4 in a 4-point scale), *B)* residual brown liquid or suction able semisolid stool (score of 2) in at least one segment; *C)* partially removable stool preventing complete mucosal visualization (score of 1) in at least one segment; *D)* at least one segment not examined due to presence of solid stool (score 0). | Grade A or B | Ell, 2008 |
|  | *Excellent:* entire mucosa seen well with no stool or liquid; *Good:* entire mucosa seen well but small fragments of stool and liquid; *Fair:* portions of the mucosa seen well but other areas not seen well due to stool and liquid; *Poor:* unprepared areas unable to see mucosa due to stool and liquid | “Excellent” and “good” preparation | Chambers, 2016 |

| **Supplemental Material C Table 2**. Assessment of secondary endpoints | | | | | | | |
| --- | --- | --- | --- | --- | --- | --- | --- |
|  | Author, year | **Acceptance of preparation strategies** | **Patients receiving adequate preparation** | **Willingness to repeat colonoscopy** | **Adverse Events** | **Hospital stay (days)** | **Repeat colon examinations** |
| Educational interventions | Chorev, 2006 | - | - | - | - | - | Repeated colonoscopies (number of inpatients) due to inadequate preparation |
|  | Rosenfeld, 2010 | - | - | - | - | - | - |
|  | Lee, 2015 | Compliance with the preparation instructions was classified as high >50% or low< 50% | > 80% of purgative ingested | Indicated with a ‘‘yes’’ or ‘‘no’’ response | Nausea, vomiting, abdominal pain, headache, electrolyte imbalance | - | - |
|  | Ergen, 2016 | - | - | - | - | Time spent (days) in the hospital after colonoscopy was completed | Repeated colonoscopies (number of inpatients) due to inadequate preparation |
|  | Chambers, 2017 | - | at least 50% of preparation ingested | - | - | - | - |
|  | Shah-Khan, 2017 | - | - | - | - | - | - |
| Bowel Regimens Modification | Seinela, 2017 | Patients were asked about their tolerance of preparation just before colonoscopy (1 = extremely unpleasant, 2 = slightly unpleasant, 3 = moderately easy and 4 = easy) | receiving 100% of preparation | Indicated with a ‘‘yes’’ or ‘‘no’’ response | Nausea, dizziness, abdominal pain, electrolyte imbalance | - | - |
|  | Reilly, 2004 | - | - | - | - | - | - |
|  | Muller, 2007 | On the day of the exam, the patient answered a structured self-assessment questionnaire - Visual Analog Scale of 100 mm where zero meant not feeling any discomfort of the symptom in question and 100 being the worst possible | - | Indicated with a ‘‘yes’’ or ‘‘no’’ response | nausea, vomiting, abdominal pain and distension | - | - |
|  | Ell, 2008 | Acceptability of the regimen was assessed by patients using visual analogue scores (VAS) ranging from 0 (excellent) to 100 (very bad) | at least 75% of the solution ingested | - | malaise, nausea, abdominal pain, vomiting | - | - |
|  | Kotwal, 2014 | Patients were asked about their tolerance of preparation just before colonoscopy (easy/fairly easy - difficult) | at least 75% of the solution ingested | Indicated with a ‘‘yes’’ or ‘‘no’’ response | Nausea, dizziness, abdominal pain, | - | - |
|  | Yang, 2015 | - | at least 75% of the solution ingested | Indicated with a ‘‘yes’’ or ‘‘no’’ response | nausea, vomiting, abdominal pain | - | - |
|  | Tae, 2015 | Patients were asked about their tolerance of preparation just before colonoscopy (good, acceptable and disgusting) | 100% of the solution ingested | Indicated with a ‘‘yes’’ or ‘‘no’’ response | nausea, vomiting, cramping, stomach bloating, dizziness, headache, sleep disturbance | - | - |
|  | Song, 2017¥ | Patients were asked about their tolerance of preparation just before colonoscopy | - | - | Nausea, dizziness, abdominal pain, electrolyte imbalance | - | - |
|  | Yadlapati, 2017 | - | - | - | - | Hospital days following preparation initiation to time of discharge | Repeated colonoscopies (inpatient or outpatient within 3 months) due to an inadequate preparation |
|  | Pontone, 2018 | A questionnaire investigating the patient’s tolerance and adherence to the protocol was administered |  |  | Nausea, vomiting, abdominal pain | Hospital days to reach a valid diagnosis |  |
| Other | Barclay, 2013 | Patients were asked about their tolerance of preparation just before colonoscopy (easy/slightly difficult/moderately difficult/extremely difficult/unable) | completing 100% of the preparation | - | - | - | - |

| **Supplemental Material C Table 3.** Risk of bias assessment of included randomized studies and authors’ judgement | | | | | | |
| --- | --- | --- | --- | --- | --- | --- |
| **Domain** | **Author’s judgement** | **Support for judgement** | **Author’s judgement** | **Support for judgement** | **Author’s judgement** | **Support for judgement** |
|  | **Ergen, 2016** | | **Seinella, 2003** | | **Muller, 2007** | |
| **Random sequence generation (selection bias)** | Low risk | Random number generator used. | Unclear risk | Exact method of randomization not described. | Unclear risk | Each patient was asked to pick an envelope including the allocation group. |
| **Allocation concealment (selection bias)** | Unclear risk | Exact concealment not described. | Unclear risk | No data provided regarding allocation concealment. | Low risk | Sealed envelopes were used. |
| **Blinding of participants and personnel (performance bias)** | Unclear risk | Patients were aware of intervention provided, but endoscopists were blinded to it. | Unclear risk | Patients were aware of preparation provided, but endoscopists were blinded to patients' allocation. | Unclear risk | Patients were unblinded to preparation received, but endoscopists were unaware of it. |
| **Blinding of outcome assessment (detection bias)** | Low risk | Endoscopists blinded to patients’ allocation | Low risk | Endoscopists blinded to patients' allocation. | Low risk | Endoscopist was unaware of patient's allocation group. |
| **Incomplete outcome data (attrition bias)** | Low risk | Attrition and exclusions provided. | Unclear risk | No data regarding attrition and exclusions. | Low risk | Attrition and exclusions are described. |
| **Selective reporting (reporting bias)** | Low risk | Outcomes reported as pre-defined. | Low risk | Outcomes reported as pre-defined. | Low risk | Outcomes reported as pre-defined. |
|  | **Ell, 2008** | | **Kotwal, 2014** | | **Tae, 2015** | |
| **Random sequence generation (selection bias)** | Low risk | Method used to generate the allocation sequence presented in sufficient detail. | Low risk | Computer generated randomization was used. | Unclear risk | No data regarding exact randomization method. |
| **Allocation concealment (selection bias)** | Unclear risk | Exact concealment method was not described. | Low risk | Sealed envelopes were used. | Unclear risk | No data regarding allocation concealment. |
| **Blinding of participants and personnel (performance bias)** | Unclear risk | Personnel blinded, but patients -although unaware- received two different preparations with different dosage and flavor. | Unclear risk | Participants were aware of allocation (morning-only vs. split). However, endoscopist was unaware of the group assignment. | Unclear risk | Patients were aware of preparation provided, but endoscopists were blinded to patients' allocation. |
| **Blinding of outcome assessment (detection bias)** | Low risk | All physicians involved in outcome assessment were unaware of the provided intervention. | Low risk | Physician was unaware of patient's allocation. | Low risk | Endoscopists were blinded to patients' allocation. |
| **Incomplete outcome data (attrition bias)** | Low risk | Attrition and exclusion were reported in details. CONSORT diagram provided. | Low risk | Attrition and exclusions described in detail. Flow diagram also provided. | Low risk | Exclusion described. |
| **Selective reporting (reporting bias)** | Low risk | All outcomes reported as pre-defined. | Low risk | Primary and secondary outcomes reported as pre-defined. | Low risk | Outcomes reported as pre-defined. |
|  | **Pontone, 2018** | | **Barclay, 2013** | |  | |
| **Random sequence generation (selection bias)** | Unclear risk | Exact randomization method not described. | Low risk | Method used to generate the allocation sequence presented in sufficient detail. |  |  |
| **Allocation concealment (selection bias)** | Unclear risk | Exact allocation concealment not described. | Low risk | Concealment method described sufficiently. |  |  |
| **Blinding of participants and personnel (performance bias)** | Unclear risk | Patients were not blinded to preparation provided. No data regarding endoscopist's blindness. | High risk | Physician and patient were aware of allocation and intervention. |  |  |
| **Blinding of outcome assessment (detection bias)** | Unclear risk | No data regarding endoscopist's blindness to patients' allocation. | High risk | Physician were aware of intervention. |  |  |
| **Incomplete outcome data (attrition bias)** | Low risk | Attrition and exclusions described sufficiently. | Low risk | Primary and secondary endpoints assessed in completeness. Exclusions described. |  |  |
| **Selective reporting (reporting bias)** | Low risk | All outcomes reported as pre-defined. | Low risk | Outcomes reported as pre-defined. |  |  |

| **Supplemental Material C Table 4**. Risk of bias assessment of included observational studies using Ottawa-Newcastle scale | | | | | | | | | | |
| --- | --- | --- | --- | --- | --- | --- | --- | --- | --- | --- |
| **Author, year** | 1. **Selection** | | | | | 1. **Comparability** | 1. **Outcome** | | | **Score** |
|  | **Representativeness of exposed cohort** | **Selection of non-exposed cohort** | **Ascertainment of exposure** | | **Demonstration that outcome of interest was not present at start of the study** | **Comparability of cohorts on the basis of the design or analysis** | **Assessment of outcome** | **Appropriate length of follow-up** | **Adequacy of follow up** |  |
| Chorev, 2006 | ***** | ***** | **-** | ***** | | ***** | **-** | **-** | **-** | 4 |
| Rosenfeld, 2010 | ***** | ***** | ***** | ***** | | ***** | ***** | ***** | ***** | 8 |
| Lee, 2015 | ***** | ***** | ***** | ***** | | ***** | ***** | ***** | ***** | 8 |
| Chambers, 2016 | ***** | ***** | ***** | **-** | | **-** | ***** | **-** | **-** | 4 |
| Shah-Khan, 2017 | ***** | ***** | **-** | ***** | | **-** | **-** | ***** | ***** | 5 |
| Reilly, 2004 | ***** | ***** | ***** | **-** | | **-** | ***** | **-** | **-** | 4 |
| Yang, 2015 | ***** | ***** | ***** | ***** | | ***** | ***** | ***** | ***** | 8 |
| Song, 2017 | **-** | **-** | ***** | ***** | | **-** | ***** | **-** | **-** | 3 |
| Yadlapati, 2017 | ***** | ***** | ***** | ***** | | ***** | ***** | ***** | ***** | 8 |

According to the scale`s “star system”, each study receives a score based upon three different perspectives: study selection groups; comparability; and the ascertainment of outcome of interest.

1. ***Selection***
2. Representativeness of intervention cohort—a] Selection from population or hospital*; b] selected from any gastroenterology department*; c] only selected group of patients; d] no description of inclusion/exclusion criteria
3. Selection of non-intervention cohort—a] drawn from same community as intervention cohort*; b] drawn from different source; c] no description of the derivation of the non-intervention cohort
4. Ascertainment of intervention for improving bowel preparation outcome—a] health record*; b] structured interview*; c] written self-report; d] no description
5. Demonstration that outcome was not present at start of study—a] yes*; b] no
6. ***Comparability***

Comparability of cohorts on basis of design or analysis—a] study controls*; b] study controls for any additional factors*

1. ***Outcome***
2. Assessment of outcome (adequate bowel preparation) —a] independent blind assessment*; b] record linkage*; c) self-report; d] no description
3. Appropriate length of follow-up —a] yes (median duration of follow up > 1 month)*; b] no
4. Adequacy of follow up—a] complete follow up*; b] minimal loss to follow up (≤20%) all ages included, all diseases, or description of those lost suggesting no difference from those followed*; c] follow up rate < 80% and no description of losses to follow up or description suggesting differences from those followed ; d] no statement

| **Supplemental Material C Table 5**. Educational Interventions and controls groups as described in the included studies | | |
| --- | --- | --- |
|  | **Intervention group** | **Control group** |
| Chorev, 2006 | Patients were given preparation as described in the control group; in addition specific guidelines in both oral and written forms were given to physicians and nurses, lectures and instruction on preparation for colonoscopy. | Patients >75 years or with moderate to severe heart or kidney failure were given 3L PEG or sodium phosphate 2 bottles of 45 mL each, to be taken with 12 glasses of tap water, the evening before the examination. |
| Rosenfeld, 2010 | Patients in this group received additionally to preparation as described in the control group, a 5 min talk during which they were instructed not to eat solid food for 24 h and they were informed of the importance of consuming as much of the preparation as possible. Gastroenterology residents used the written materials as a guide but did not necessarily strictly read the instructions to the patient. Patients were given the opportunity to have their questions answered at the end of the counselling session. The gastroenterology resident provided the counselling session at the bedside where the written instructions were reviewed and left with the patient. Nursing staff was not given any specific instructions and left to provide their ‘usual care’. Patient’s family was not involved. | Clear liquid diet for 24 h before colonoscopy, withhold of food and fluids after midnight on the day of the procedure, and 4 L of PEG over a span of 4 h to 6 h starting at approximately 12:00 the day before the procedure. Patients were told that they would undergo a colonoscopy and that they would need to take a laxative solution the day before in preparation. |
| Lee, 2015 | Patients received preparation as described in the control group along with explanation of steps required for bowel preparation given by intensively educated nurses. Expert endoscopists provided enhanced education, consisting of a leaflet and a lecture, to nurses who belonged to the educated ward. The enhanced education included importance of bowel preparation, possible adverse effects and methods to reducing a patient’s anxiety. Also pre-colonoscopy diet and the rationale for this diet, instructions for completion, and importance of drinking additional water. The leaflet was placed on the wall of the educated ward, and review training sessions were repeated. | Patients received the standard explanations from nurses who had received no enhanced education. No additional education was provided to nurses who were assigned to the control ward.  Bowel prep consisted of 2 liters PEG + ascorbic acid administrated either as split or full dose. |
| Chambers, 2016 | Patients received preparation as described in the control group according to electronic medical record system on colonoscopy preparations and procedures. The in-service education took about 1 hour and included time for questions and answers. At the request of the nurses, copies of the presentation with color pictures were shared for use when teaching the patients and their families. When patients arrived in the endoscopy suite for colonoscopy, they were asked to answer questions related to the bowel preparation and general knowledge about the procedure | Patients received the standard explanations from nurses. No additional education was provided  Bowel prep consisted of PEG solutions. |
| Ergen, 2016 | Patients received preparation as described in the control group along with an educational booklet before colonoscopy. The booklet was delivered to the inpatient nurse prior to 6pm the evening before the colonoscopy. Primary investigator observed the nursing staff delivering the booklet to the patient’s room without interacting. The “bundling” of bowel preparation and booklet together suggested usual care to the patients. | Patients received a standard bowel preparation: clear liquid diet the day prior to procedure followed by split dose PEG with electrolytes. Instructions for bowel preparation include an order set that delineates the exact time the patient should start consuming bowel preparation. Patients were instructed to consume 2 L between 6pm and 8pm the night prior to colonoscopy, and 2 L between 5am and 7am on the day of colonoscopy. |
| Shah-Khan, 2017 | Patients received preparation as described in the control group along with education by medical residents and nursing staff, involving both speaking to them individually and distribution of a handout. The specifics of the preparation process were explained as well as various troubleshooting steps. Patient were given a handout explaining why a successful bowel preparation is important. | Patients received the standard explanations from nurses. No additional education was provided. Bowl preparation consisted of PEG solutions. |

**Supplemental Material D Figure 1**

**
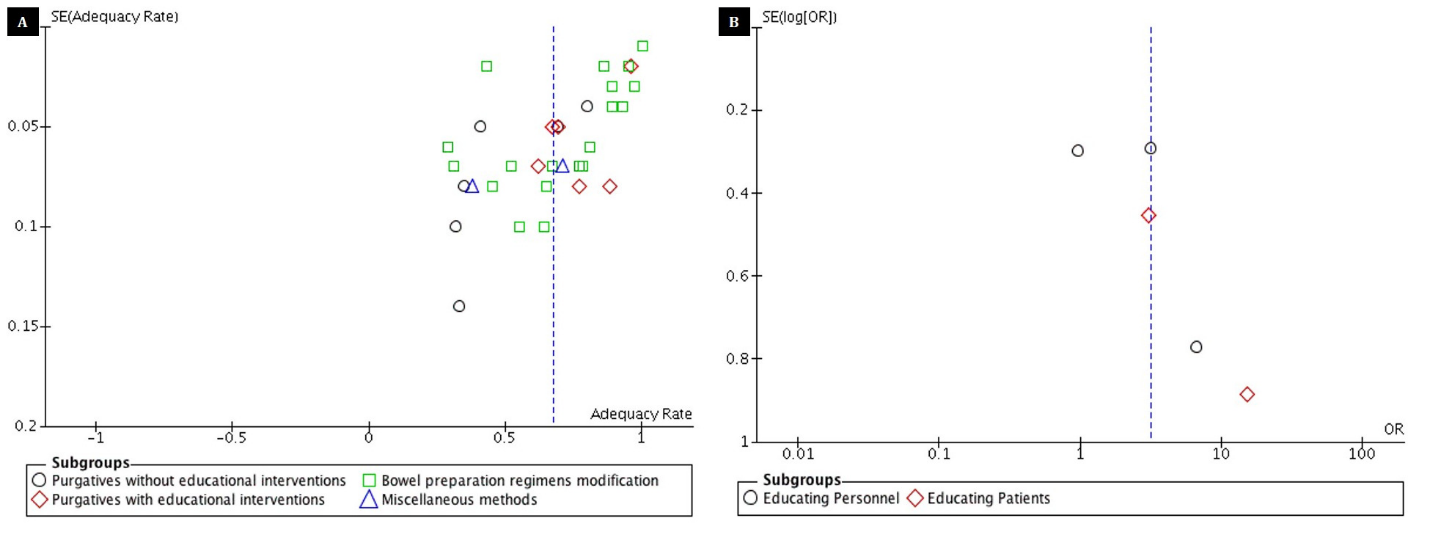
**

**Supplemental Material D Figure 2**

**
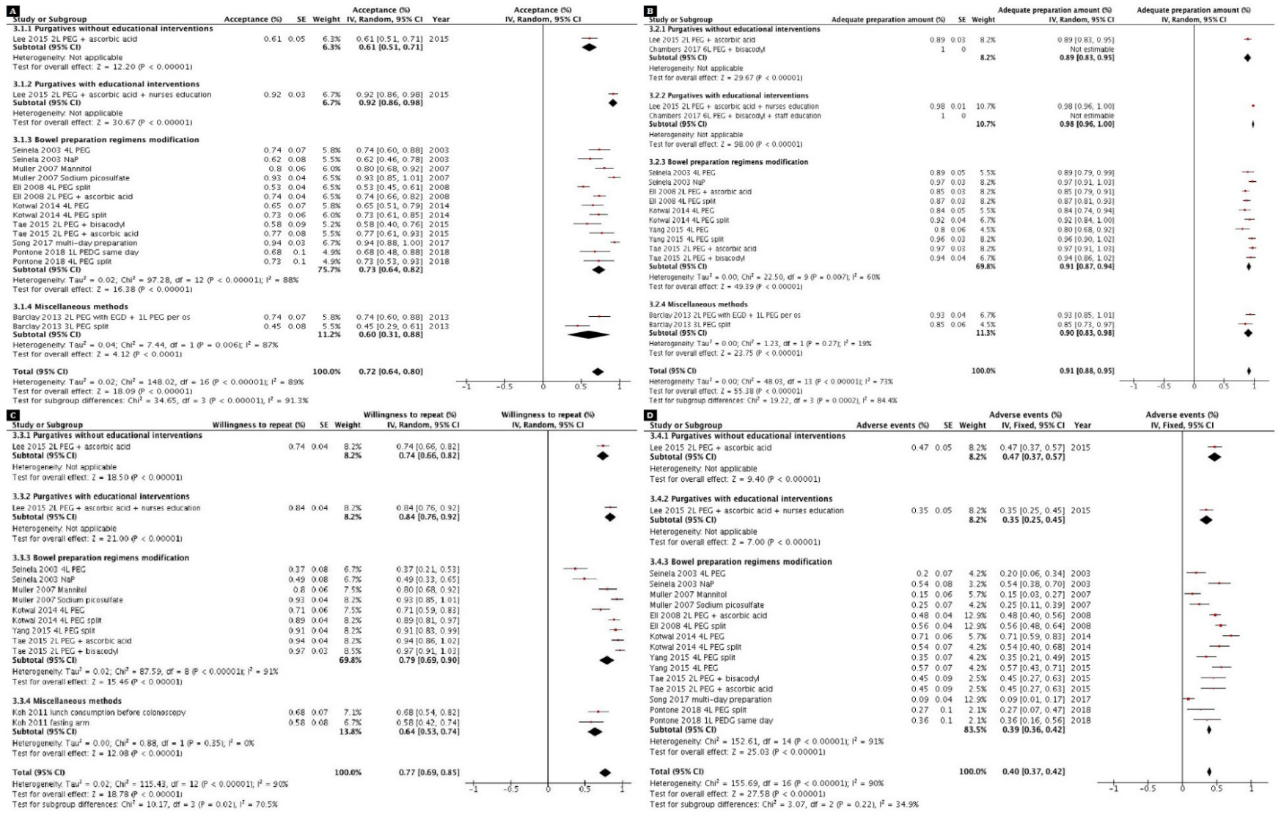
**
